# Synchrotron Infrared Microspectroscopy for Stem Cell Research

**DOI:** 10.3390/ijms23179878

**Published:** 2022-08-30

**Authors:** Jiang Qian, Xue Gao, Ya-Di Wang, Xue-Ling Li, Jun Hu, Jun-Hong Lü

**Affiliations:** 1 Shanghai Institute of Applied Physics, Chinese Academy of Sciences, Shanghai 201800, China; 2 Jinan Microecological Biomedicine Shandong Laboratory, Jinan 250000, China; 3 Shanghai Advanced Research Institute, Chinese Academy of Sciences, Shanghai 201203, China; 4 University of Chinese Academy of Sciences, Beijing 100049, China; 5 School of Pharmacy, Binzhou Medical University, Yantai 264003, China; 6 Collaborative Research Center, Shanghai University of Medicine & Health Sciences, Shanghai 201318, China

**Keywords:** synchrotron infrared microspectroscopy, stem cell differentiation, pluripotent stem cells, cancer stem cells

## Abstract

Stem cells have shown great potential functions for tissue regeneration and repair because of their unlimited self-renewal and differentiation. Stem cells reside in their niches, making them a hotspot for the development and diagnosis of diseases. Complex interactions between niches and stem cells create the balance between differentiation, self-renewal, maturation, and proliferation. However, the multi-facet applications of stem cells have been challenged since the complicated responses of stem cells to biological processes were explored along with the limitations of current systems or methods. Emerging evidence highlights that synchrotron infrared microspectroscopy, known as synchrotron radiation-based Fourier transform infrared microspectroscopy, has been investigated as a potentially attractive technology with its non-invasive and non-biological probes in stem cell research. With their unique vibration bands, the quantitative mapping of the content and distribution of biomolecules can be detected and characterized in cells or tissues. In this review, we focus on the potential applications of synchrotron infrared microspectroscopy for investigating the differentiation and fate determination of stem cells.

## 1. Introduction

Stem cells are undifferentiated or incompletely differentiated cells that can produce daughter cells with high proliferative potential [1]. Until now, precise regulations of differentiation and fate determination for stem cells still need further investigations. Because cell differentiation is highly dynamic with certain molecular profiles, multiple methods have shown the inevitable limitations in providing the analysis of differentiation, which includes quantitative real-time polymerase chain reaction, western blot, and immune-fluorescent histochemical staining [2]. By these techniques, specific genes or proteins, or organelles were detected. However, the content of carbohydrates, lipids, and nucleic acids in cells cannot be analyzed. Hence, the development of a novel method or system is needed.

Synchrotron infrared microspectroscopy, known as the synchrotron radiation-based Fourier transform infrared microspectroscopy (SR-FTIR), is a more powerful and effective tool with higher resolution and improved signal-to-noise ratio compared to traditional FTIR with a global source [3,4]. SR-FTIR can simultaneously detect discrete changes at the cellular and sub-cellular levels. With a continuous light source, synchrotron radiation infrared covers near-infrared, middle infrared, and far infrared regions, which can provide much more information for the biological samples [5]. Vibrational and rotational transitions are involved in this spectroscopy with low energy. By measuring the wavelength range, peak areas, and spectrum intensities, the functional molecular structures in cells or tissues can be well characterized without damage [6]. Based on the outstanding focusability, stability, and polarization, the technology has been widely applied to analyze complex components in biological samples. In addition, chemical information within the cellular dimension can be acquired by SR-FTIR imaging [7,8].

This article begins with a brief introduction to SR-FTIR spectra and imaging, as well as the general statistical and analytical methods for stem cell investigation [9,10,11,12,13]. Next, we reviewed the application of SR-FTIR in four typical differentiation processes, including adipogenic, osteogenic, chondrogenic, and neurogenic differentiations, which were essential for maintaining homeostasis in mammals [14,15,16,17,18]. In addition, the potential of SR-FTIR was summarized in multiple parts, including characterization and localization of stem cell niches in tumors [19,20,21,22,23,24,25,26], as well as the investigation of the intricate chemical structures of stem cells in normal adult tissues [27,28,29,30,31,32]. Moreover, other studies, such as on liver regeneration and senescence, were also listed [28,33,34,35,36,37]. Overall, we focus on the main application of SR-FTIR in providing detailed information on the differentiation and fate determination of stem cells [38,39].

## 2. Brief Overview of Synchrotron Infrared Microspectroscopy in Stem Cell Researches

**Figure 1:**
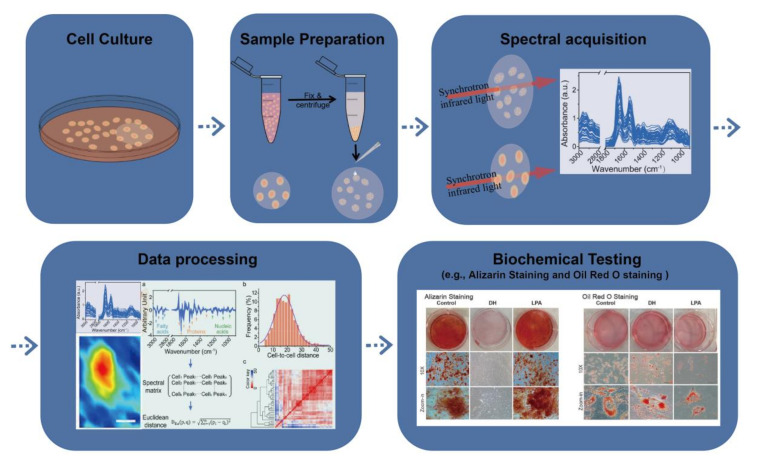
A representative workflow for SR-FTIR study of stem cells [11,17]. The Figure in “Spectral acquisition” was reproduced from Figure 1b in reference [11], Figures of “Data processing” were reproduced from Figure 1a and Figure 2a–c in reference [11]. The small Figures in “Biochemical testing” were reproduced from Figure 3a,c in reference [17].

A representative workflow for the SR-FTIR study of stem cells (Figure 1) usually started with various treatments amongst cells and obtained infrared spectra with plenty of details. Multiple statistical methods, such as histological and immune-histological staining, were typically applied in those researches.

### 2.1. Development of Synchrotron-Based FTIR Microscopy and Imaging

**Figure 2:**
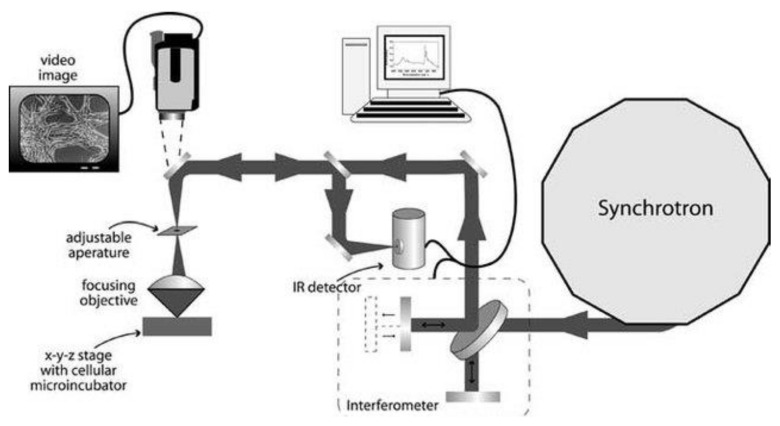
Synchrotron-based FTIR microspectroscopy setup scheme. The collected and collimated synchrotron radiation was transported to an FTIR interferometer bench. Then the modulated infrared beam was focused onto samples. The reflected light from samples was collected and recorded using an IR detector. All infrared spectra data and information were processed by computers [41].

Synchrotron source coupled with Fourier transform infrared microspectroscopy and highly-sensitive infrared imaging detectors, as an effective and practical imaging method, can be applied to investigate the biomolecular structure information with cellular and sub-cellular resolution [9]. For decades, chemical imaging of living cells has been investigated, which according to the vibration spectroscopy in fatty acids, proteins, or nucleic acids components were able to construct the chemical image of the cells [10,40]. The setup of SR-FTIR was given in the Scheme (Figure 2). The list of major infrared absorptions in SR-FTIR of stem cells is presented in Table 1.

### 2.2. Data Processing of Synchrotron-Based FTIR Microscopy and Imaging

The identification and characterization of molecular spectral features in raw infrared spectra has been a difficult problem because vibrational bands were mainly superimposed on light scattering and background noise. The basic data processing procedure of infrared spectra consisted of smoothing, baseline correction, and normalization, followed by multivariate statistical methods. Moreover, a strong scatter may appear when radiation wavelengths regions from 3 to 10 mm and the size of samples were around 1 to 10 μm. Mie scattering was applied to correct the distorted spectrum and displayed the pure absorbance spectrum [42].

Multivariate statistical approaches were applied to analyze SR-FTIR spectra, such as visual observation-related methods, unsupervised statistical methods, and supervised statistical methods [43,44]. For visual observation-related methods, second-derivative and deconvolved spectra were widely applied, which not only eliminated broad baseline slopes in the original infrared spectrum but also made the overlapping spectra resolvable. However, with the drawback of a low signal-to-noise ratio, visual observation-related methods were usually coupled with unsupervised or supervised statistical methods.

Principal component analysis (PCA) and unsupervised hierarchical cluster analysis (UHCA) were typical statistical procedures. In PCA, a large number of complex variables had been simplified and displayed the variance of infrared spectra information. Most directions of vital variation could be illustrated by the first two or three PCs factors, which were applied in multiple articles with a two or three-dimensional diagram [45,46]. In UHCA, the infrared data sets were performed using Ward’s algorithm, identifying two similar infrared spectra using a matrix defining the distance between the spectra [47].

As for supervised statistical methods, partial least squares discriminant analysis (PLS-DA) was widely applied in predicting and quantifying chemical compositions [7,38]. During human embryonic stem cells (hESCs) differentiation, PLS-DA was used to predict Y values which indicate the 95% confidence interval. PLS-DA scores and loading plots were examined to determine the infrared spectral signatures of experimental and control groups at each time interval.

Distance measurements were performed in many algorithms in unsupervised or supervised statistical methods. Euclidean distances or the squared Euclidean distances were calculated to detect the heterogeneity of the biochemical profiles of samples. The cell-to-cell similarity was negatively correlated with the value of the Euclidean distance, which means that high similarity was indicated by smaller distances.

The spatial distribution for chemical components of cells was generally based on specific infrared absorption bands [13]. The highly complex infrared data were transformed into lower-dimensional space using PCA with second-derivative processing. The cell-to-cell Euclidean distances were calculated to verify sample-to-sample similarity.

## 3. Stem Cell Differentiation

Stem cell differentiation, such as adipogenic, osteogenic, chondrogenic, and neurogenic differentiation, are intricate and well-programmed processes [48,49,50]. Representative biomarkers of differentiation steps are limited, especially in the early stage. Thus, a label-free and sensitive vibrational spectral method is urgently needed.

For instance, during adipogenic differentiation of mesenchymal stem cells (MSCs), 14 key specific absorption bands were selected based on the minimum points of second-derivative spectra among fatty acids (2940–2910 cm^−^^1^), proteins (1670–1600 cm^−^^1,^), and nucleic acids (1133–1033 cm^−^^1,^) [11]. The whole spectra or sub-regional spectra were applied to calculate the cell-to-cell distance. After treating the adipogenic culture medium, Euclidean distances were analyzed and displayed in distribution histograms, indicating the heterogeneous changes of MSCs.

In studying vibrational signatures among cells, a certain number of cells were required to differentiate the measured signal from control groups with a 95% confidence interval. Wang and colleagues developed a novel statistical method and procedure to quantitatively evaluate the single-cell spectral variation of different cell lines and cellular states [12]. Infrared spectral data were subjected to PCA to investigate the spectral changes in a lower-dimensional space. In this study, 16 specific infrared absorption peaks were analyzed. Based on the coefficient of variation of the Euclidean distance and Chebyshev distance, 20 cells were required to obtain the same trend of batch effect in MSCs.

### 3.1. Adipogenic and Osteogenic Differentiation

**Figure 3:**
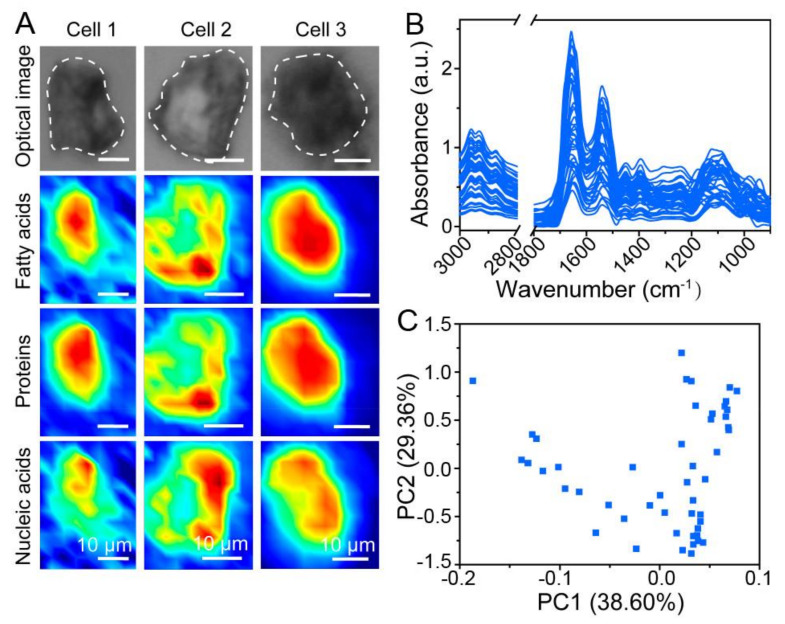
Infrared phenotypic heterogeneity of MSCs during adipogenic differentiation. (**A**) Single-cell optical images and chemical complexity maps of MSCs in the spectral regions associated with fatty acids, proteins, and nucleic acids. (scale bars: 10 μm) (**B**) Infrared spectra after baseline correction and vector normalization at the wavenumber 3000–800 cm^−1^. (**C**) Score plots of PC1 and PC2 after PCA based on the second-derivative spectra in (**B**) [11].

During early adipogenic differentiation, an initial vibrational spectroscopy study of human mesenchymal stem cells (hMSCs) showed lipids and nucleic acids related regions changed dramatically at the single-cell level [16]. Before obtaining SR-FTIR spectra, hMSCs cells were treated with adipogenic medium for days and fixed in formalin. In this study, biological macromolecule structural changes have taken place during the whole process of adipogenesis from 0 to 21 days. All SR-FTIR spectra data were normalized by Resonant Mie scattering extended multiplicative correction (RMie-EMSC) and evaluated by PCA in combination with linear discriminant analysis (PCA-LDA). Lipids (2900–2800 cm^−1^ and 1470–1300 cm^−1^), proteins (1700–1500 cm^−1^), and nucleic acids (1200–1000 cm^−1^) were processed, together with the peaks at 1398 cm^−1^ and 1072 cm^−1^, corresponding to lipids and nucleic acids separately. Adipogenesis-induced and non-induced cells could be distinguished on the first day using the PCA-LDA score plot (Figure 3c).

Previous research carried out by Lorthongpanich, in which SR-FTIR was applied to investigate the Yes-associated protein (YAP), an important effector protein in both osteogenic differentiation and adipogenic differentiation [17]. In this study, YAP-knockdown and YAP-overexpressing hMSCs cells were investigated. The mean SR-FTIR spectra data of hMSCs were analyzed in lipids regions (3000–2800 cm^−1^), protein regions (1700–1500 cm^−1^: amide regions I and II), nucleic acids regions, and carbohydrate regions (1300–900 cm^−1^). Spectra regions at 2921 cm^−1^, 2850 cm^−1,^ and 1741 cm^−1^ from lipids (CH_3_, CH_2,_ C=O stretching) indicated the highest positive loading plot from PC1. As for PC2, positive loading can be defined by nucleic acids (1238 cm^−1^) and carbohydrate regions (1035 cm^−1^). The results suggested that a lack of YAP could promote adipogenic differentiation.

**Figure 4:**
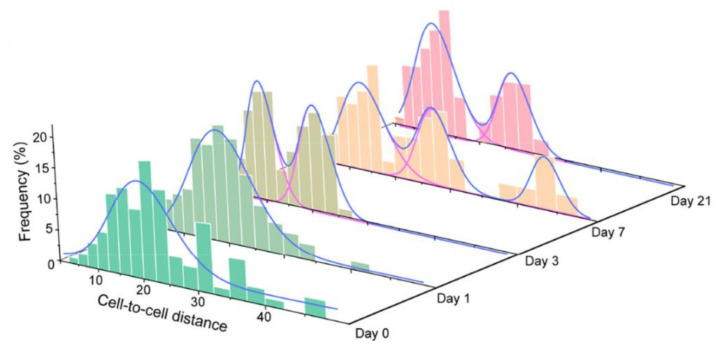
The histograms and Gaussian-fitted curves of intercellular Euclidean distances under adipogenic medium. Red violet lines: isolated peaks; blue lines: peak sums [11].

More recently, for quantitatively assessing cellular heterogeneity, a promising strategy was developed based on single-cell synchrotron FTIR microspectroscopy and computational approaches [11]. In this research, data were obtained by SR-FTIR, and cell-to-cell similarity distances were calculated. During adipogenic differentiation of MSCs cells the dynamic heterogeneity changes have been investigated. As shown in Figure 3a, the chemical mapping images of different single cells were shown in fatty acids, proteins, and nucleic acids. Besides, the distribution of cell-to-cell Euclidean distances of MSCs was shown, and the heterogeneity changes were revealed clearly (Figure 4). On the second day, the existence of two subpopulations suggested the potential membrane structure changed rapidly. Moreover, the variation of the protein region started on the third day and lasted for the entire experiment.

### 3.2. Chondrogenic Differentiation

SR-FTIR is a powerful method to investigate cellular components induced hMSCs in chondrogenic differentiation [14]. There was no significant difference between control and chondrogenic differentiation-induced hMSCs during the first 7 days at both mRNA and protein levels. Although minor changes appeared at the transcriptional level, whether it was triggered by the refreshment or induction of the culture medium remains unclear. Based on the infrared spectra, unique characteristics of lipids, proteins, and nucleic acids were detectable in the early induction days (7 days) of chondrogenic differentiation, especially the protein-related bands. The average spectra were obtained after analyzing about 100 spectra; collagen-related differences were revealed in amide III (near 1338 cm^−1^), P-O stretching (1230 cm^−1^), and C-O-C stretching (1203 cm^−1^), as well as proteoglycan proteins correlated spectra differences in S-O stretching (1245 cm^−1^) and C-O-C stretching (1175–960 cm^−1^). Meanwhile, opposite correlations were revealed between aggrecan (1338 cm^−1^ and 1245 cm^−1^) and collagen type II (1175–950 cm^−1^) by the average second derivative spectra.

### 3.3. Neurogenic Differentiation

Neural differentiation was studied by FTIR spectroscopy in mouse embryonic stem cells (mESCs) [18]. Focal plane array-based Fourier-transform infrared (FPA-FTIR and SR-FTIR were applied to distinguish the early or late stage of neural differentiation of mouse embryonic stem cells. Embryoid bodies (EBs), neural progenitor cells (NPCs), and embryonic stem-derived neural cells (ESNCs) were investigated by FTIR, then obtained spectra were processed by PCA and HUCA. According to the advantages of these two FTIR spectroscopy methods, clumps of cells were studied by FPA-FTIR, and single cells were investigated by SR-FTIR. The absorption bands increased significantly at 2958, 2922, and 2851 cm^−1^ (ν_as_ CH_3__,_ ν_as_ CH_2,_ and ν_s_ CH_2_ stretching vibrations, respectively), which were related to lipids during the time of neural differentiation, especially glycerophospholipids. During differentiation of NPCs and ESNCs, the rising bands of amide I at 1659 and 1637 cm^−1^ indicate α-helix structures increased and β-sheet secondary structures decreased.

Recent research pointed out that the functional polymer scaffolds promoted the repair of neural tracts in the central nervous system. In this study, the morphology and surface chemistry of electrospun polyester fibers were studied by SR-FTIR at 3050–2800 cm^−1^ (the lipids) and 1800–1400 cm^−1^ (amide I–II regions) for two days [15]. In the amide I band (1700–1600 cm^−1^), a decrease of α-helix structures (1657 cm^−1^) was noticed, and an increase of β-sheet structures (1693 cm^−1^) was observed, which were related to antiparallel β-sheet and β-turns structures. And the results of the amide II region (1516 cm^−1^) were consistent with the results of the amide I region. The infrared results demonstrate that specific polymer scaffolds fiber showed a wide influence on the production of NPC differentiation.

## 4. Cancer Stem Cells

The aggressiveness of cancer is closely related to the content of cancer stem cells. That is, the higher the cancer stem cell content, the more aggressive the cancer [51]. Cancer stem cells may promote the proliferation of tumors, and are also a fragile point of cancer treatment. There is a lack of universal biomarkers to identify cancer stem cells amongst tumors; hence label-free and non-invasive approaches are urgently needed.

Glioblastoma is the most common and malignant primary tumor of the central nervous system, which possesses a high capacity for self-renewal [24]. Previous research showed that FTIR-based methods could estimate the content of cancer stem cells in glioma. Based on the difference of nucleic acids related regions, they revealed that carbohydrate metabolism was affected by all-trans retinoic acid-induced differentiation, which was basically based on bands around 1300–1200 cm^−1^ (nucleic acids) and bands around 1120–940 cm^−1^ (C-O linkages of cellular sugars) [22,23].

SR-FTIR is a useful method to investigate drugs and radiation resistance of glioblastoma cells. The biochemical changes of complex biomolecules interacting with boron clusters were investigated by SR-FTIR coupling with single element detectors. Nuez-Martinez and colleagues demonstrated that two phenotypes of glioma stem-like initiating cells, radioresistant mesenchymal PG88 cells and proneural glioma stem-like initiating cells7 (GIC7), could be studied according to variations in lipids, proteins, and nucleic acids. These two cells were modified by Na[o-COSAN], and spectra changes in lipids (2966 cm^−1^ and 2922 cm^−1^) frequencies were induced in PG88 cells.

**Figure 5:**
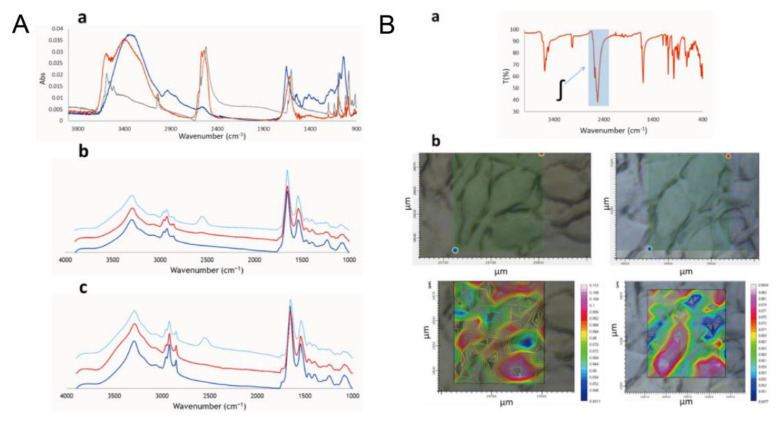
Na[o-COSAN]^−^ uptake analyses and microscopy images of GICs. A: GICs Na[o-COSAN]^−^ uptake analyses by SR-FTIR. (**A)** (**a**) Strong bands of ν(B-H) at 2582–2522 cm^−1^ and ν(C _c__luster_-H) at 3031 cm^−1^ were detected. Gray: Na[o-COSAN] in solid-state; Orange: Na[o-COSAN](2 mM) in aqueous solution; Blue: Na[o-COSAN](2 mM) in culture media solution. (**b**) IR spectra of GIC7 cells. Dark blue: control group; Red: Cells were incubated in Na[o-COSAN](200 µM)-culture medium for 5 h. Light blue: Cells were incubated in Na[o-COSAN](2 mM)-culture medium for 5 h. (**c**) IR spectra of PB88 cells. Dark blue: control group; Red: Cells were incubated in Na[o-COSAN](200 µM)-culture medium for 5 h. Light blue: Cells were incubated in Na[o-COSAN](2 mM)-culture medium for 5 h. B: Microscopy images and mappings of GIC7 cells after being treated with Na[o-COSAN](2 mM) for 5 h. (**B)** (**a**) The ν(B-H) signals bands at 2620–2460 cm^−1^. (**b**) Corresponding maps of two different areas of culture cells. The intensity of the red color indicates the nuclear localization of Na[o-COSAN] [26].

Compared to the infrared spectra of raw Na[o-COSAN] compounds in H_2_O and culture medium, the different bands of ν(B-H) at 2582–2522 cm^−1^ and ν(C cluster-H) at 3031 cm^−1^ were detected. The small peaks in the culture medium group of ν(B-H) may be correlated with the interaction between Na[o-COSAN] and the biomolecules in the culture medium (Figure 5A(a)). After treating cells in a 200 µM Na[o-COSAN] mixed culture medium for 5 h, the B-H stretching peaks were observed at 2557 cm^−1^ and 2537 cm^−1^ in both PG88 and GIC7 cells (Figure 5A(a),(b)). Besides, further study revealed that the Na[o-COSAN] compounds were clustered in the nucleus (Figure 5B) [26].

X-ray irradiations treated glioma cells were studied using SR-FTIR [25]. After irradiation, vibrational changes can be observed, associated with DNA damage and cell death. Specifically, the bands of CH_2_ and CH_3_ (3100–2800 cm^−1^), and the bands of PO_2_^−^ symmetric and PO_2_^−^ asymmetric (1238 cm^−1^), changed obviously.

Ahmadzai et al. [21] found the differentiation process from putative stem cells to transit-amplifying cells and ended to terminally differentiated cells by SR-FTIR. The data of SR-FTIR were analyzed using linear discriminant analysis or PCA in the study of cancer stem cell differentiation.

Paneth cells, transit-amplifying cells, terminally differentiated cells, and multipotent stem cells with distinct base regions can be distinguished between normal or adenocarcinoma human intestines. Most stem-like cells were segregated by the chemical-bond vibrations of proteins and nucleic acids. In a normal small intestine, the bands at 1680 cm^−1^ (amide I) and 1080 cm^−1^ (ν_s_PO_2_^−^) were identified as biomolecular markers for stem cells. In a normal colon, the main biomarkers of stem cells were the bands at 1500–1550 cm^−1^ and 1180 cm^−1,^ which indicated amide II and carbohydrate separately. In small intestine adenocarcinoma, changes in stem cell-like (SC-like) cells were at 1650 cm^−1^ (amide I) and 1080 cm^−1^ (ν_s_PO_2_^−^). As for SC-like cells in colon adenocarcinoma, major variations were at 1630 cm^−1^ (amide I), 1580 cm^−1^ (amide II), 1370 cm^−1^ (protein) and 1260 cm^−1^ (ν_s_PO_2_^−^).

Stem-cell lineage in corneal squamous cell carcinoma could also be investigated by SR-FTIR. Differences were revealed among stem cells, transient-amplifying cells, terminally-differentiated cells, and squamous cell carcinoma cells. In cancerous tissue, DNA changes could be detected from the peaks of around 1225 cm^−1^, 1080 cm^−1^, and 1030 cm^−1^ [20]. Stem cells and squamous cell carcinoma could be distinguished by lipids, proteins, and RNA; terminally-differentiated cells and squamous cell carcinoma could be distinguished by DNA and RNA.

In addition, SR-FTIR spectroscopy was a useful tool in determining and characterizing putative stem-like cells between renal epithelial carcinomas and normal tissues [19]. In that study, Hughes et al. found that the infrared spectral profiles related to DNA and lipids were two key spectral signatures in distinguishing stem cell-like cells among renal epithelial carcinoma tissues.

## 5. Putative Stem Cell in Normal Cells

Adult stem cells are undifferentiated cells found in many adult tissues and organs, such as the endometrium, human intestinal crypts, corneal, and breast. SR-FTIR technology plays an important role in studying the identification and location of adult stem cells in normal tissues. The SR-FTIR-based method has been proved practical for identifying the intricate chemical structures of stem cells in adult tissues.

Epithelial stem cells, mesenchymal stem cells, and endothelial progenitor cells were three types of stem cells that could proliferate and produce daughter cells in the endometrium [52]. Although biochemical techniques have been developed to probe the rough location and activity of those stem cells, the exact location of endometrial stem cells remains elusive. Moreover, the biological specificities of endometrial stem cells were closely related to endometrial proliferative disorders, which may result in endometriosis and carcinogenesis. Based on the previous studies, putative stem cells existed in endometrial tissue, while the pinpointed location can be identified by SR-FTIR and FPA-FTIR microspectroscopy [32]. In this study, critical vibrational changes were observed in the amide I, amide II, and ν_s_PO_2_^−^, which characterized the detailed location of putative stem cells amongst the epithelial cells. Two groups of visible differences were indicated by the averaged spectra. Furthermore, it is revealed by the subtraction spectrum that four marked differences were located around 1650 cm^−1^, 1550 cm^−1^, 1390 cm^−1^, and 1080 cm^−1^ related to amide I, amide II, nucleic acids, and ν_s_PO_2_^−^ in RNA/DNA, separately.

Furthermore, to characterize the putative stem cells in human intestinal crypts, infrared spectral differences were observed between the small intestine and large intestinal crypts [27,28]. Consistent with the studies in epithelial cells, contributory factors in distinguishing putative stem cells of intestinal crypts were amide I, amide II, ν_as_PO_2_^−^, ν_s_PO_2_^−^, protein and nucleic acid phosphorylation located at the peaks around 1650, 1550, 1225, 1080, 1800-1480, and 1425-900 cm^−1^, respectively.

Adult corneal epithelial stem cells were able to proliferate and differentiate for a lifetime. Based on the biochemical signatures in human-derived corneal tissue, SR-FTIR played a serviceable role in distinguishing and localizing putative adult corneal stem cells and stem cells derived cells, such as transiently amplified (TA) cells and terminally differentiated (TD) cells [29]. Compared TA cells with TD cells, several significant differences were detected by median absorptions varying from 1714 cm^−1^ to 1225 cm^−1^. At the wavenumber of 1714 cm^−1^, the band of C=O stretching vibration in nucleic acid was detected; at the wavenumber around 1600cm^−1^, there was a sensitive zone of nucleic acid changes; the peaks around 1480 cm^−1^ to 1450 cm^−1^ were related to multiple components including lipids, proteins, and nucleic acids; the peaks around 1380 cm^−1^, 1269 cm^−1^, and 1225 cm^−1^ were linked to cell cycle and nucleic acids. In terminally differentiated cells, changes around the peaks of 1650 cm^−1^, 1120 cm^−1^, 1080 cm^−1^, and 1030 cm^−1^ were discovered, which suggested the alteration of the secondary structure of proteins and the observation of RNA, protein, and glycogen.

It was presented by Simon et al. that putative corneal SCs, TA, and TD cells could be discriminated by SR-FTIR [30]. SCs were separated from TA and TD cells according to several essential peaks, including DNA regions related to ν_s_PO_2_^−^ (1080 cm^−1^) and ν_as_PO_2_^−^ (1225 cm^−1^), as well as protein regions related to asymmetric CH_3_ (1443 cm^−1^). Additionally, major discriminating factors between TA and TD cells were amide I (1650 cm^−1^), amide II (1550 cm^−1^), and lipids (around 1740 cm^−1^).

The studies in benign human breast tissue revealed that age-related molecular changes could be detected by SR-FTIR. Based on vibrational spectroscopy, stem cells were indicated in the terminal ductal lobular epithelium [31]. When processing exploratory PCA, the first 3 PCs were calculated to distinguish putative stem cells within the myoepithelial and luminal cells. The key bio-maker in those cells was Amide I.

## 6. Stem Cells Related Regeneration

Stem cells, with their potential for multi-directional differentiation, high portability, and low immunogenicity, allows types of stem cells to serve as ideal implant material in disease therapies such as fracture treatment, cornea regeneration, neural regeneration, and liver regeneration [34].

The damaged liver could be recovered by parenchymal liver cells, which had regenerative capacity. At present, stem cell-based liver regeneration therapy is organized to deal with the persistent shortage of suitable donor organs. The transplantation of stem cells held great potential for end-stage liver disease treatment. Meanwhile, SR-FTIR could be used to investigate the early and late cell differentiation stages in hepatocytes by analyzing various spectral features.

During the differentiation of mouse embryonic stem cells into hepatocyte-like cells, Thumanu and colleagues investigated three differentiation steps which included endoderm induction, hepatic initiation, and hepatocyte-like cells. In this study, vibrational changes served as practical information in distinguishing mature hepatocyte-like cells from stem cell progenitors [36]. The characteristic absorbance results suggested that an increase of amide I band was observed around 1656 cm^−1^, indicating a predominance of α-helical protein structure in mature hepatocyte-like cells. However, compared to mature hepatocyte-like cells, the higher absorbance around 1627 cm^−1^ suggested that the β-sheet secondary structure was more intense in stem cell progenitors. In addition, compared to mature hepatocyte-like cells, higher lipid levels were revealed in hepatic progenitor cells depending on CH_3_, CH_2_, and C-H stretching bands at 2923, 2852, and 1740 cm^−1^, respectively.

SR-FTIR was a practical method not only in sorting early stages of hepatocyte differentiation in mouse embryonic stem cells but also a powerful approach for a chemical injured liver study in rat bone marrow-derived mesenchymal stem cells.

To investigate the regenerative effects of stem cell therapy regarding liver injury, biochemical changes of macromolecular composition were examined by SR-FTIR in dimethylnitrosamine (DMN) injured liver after the injection of rat bone marrow-derived mesenchymal stem cells (rBM-MSCs), rBM-MSCs derived differentiated cells (rBM-MSC-DSCs) and normal liver tissues (the group of control) [37].

Compared with the normal group (without DMN treatment) and the rBM-MSC–transplanted group, the rBM-MSC-DSC-transplanted group showed a decrease in carbohydrates regions (1190–970 cm^−1^) of liver tissue. Meanwhile, in normal liver tissue–transplanted animals, the increased infrared absorbance was observed at the band of C-O from polysaccharides. Additionally, rBM-MSC-DSCs held great potential for ameliorating liver damage according to the colocalization of normal liver and rBM-MSC-DSCs treated DMN-injured liver on the score plot.

## 7. Other Studies

Infrared spectroscopy has been widely applied as a promising method to investigate aging and regenerative medicine in stem cells [33]. Georgios and colleagues have analyzed normal human breast tissues from eleven different aged women by SR-FTIR [31]. All data revealed that SR-FTIR was a practicable tool for differentiating biomarkers and identifying temporal variation changes in breast tissue. First, the key factors in inter-individual variations were Amide I and DNA/RNA of the bands at 1630 cm^−1^ and 1080 cm^−1^, respectively. Likewise, the vital spectral signature in temporal variations was Amide II of the bands around 1456 cm^−1^. Furthermore, two critical factors in breast tissue were Amide I (the bands around 1647 cm^−1^) and ν_s_PO_2_^−^ (the bands around 1094 cm^−1^), which were closely related to temporal changes in individual cell populations.

In particular, Sandt and colleagues analyzed groups of induced pluripotent stem cells reprogrammed by different methods and identified by SR-FTIR [35]. In this study, infrared spectra of induced pluripotent stem cells (iPSCs) were evaluated, and PC loadings were suggested by spectral clusters after treating different reprogramming strategies. First, based on the single-cell vibrational spectral of iPSC, the same biochemical compositions were observed when the parental somatic amniotic fluid cells were reprogrammed by group OS (Oct4 and Sox2 transcription factors) or by group OSLN (Oct54, Sox2, Lin28, and Nanog transcription factors). Biochemical heterogeneity was also shown between iPSC and hESCs in the infrared spectra, which were largely related to the glycogen of the bands around 1030 cm^−1^. Moreover, the accumulation of glycogen was a signature of faster metabolism as well as a label of tumorigenicity.

## 8. Future Directions

In recent decades, SR-FTIR-based microspectroscopy and imaging have been widely applied in stem cell-related research. A phenotypic screening approach revealed that the chemical information in single cells could be further studied [13,53]. SR-FTIR was applied here to identify changes related to molecular biomarkers in cancer stem cells. Without the need for fluorescent or radioactive labels and with a small number of samples, SR-FTIR was a suitable tool to measure cells or tissues absent on biomarkers or labels, such as glioma stem-like initiating cells and putative stem cells in human intestinal [19,26].

As a label-free and non-invasive method with a high signal noise ratio, SR-FTIR plays an important role in disease diagnosis and treatment. For example, SR-FTIR shows a clear superiority in chemotherapy and radiotherapy [25,54]. Similarly, the potential of SR-FTIR is also highlighted in analyzing complicated responses of stem cell-related materials [55,56].

Incredible efforts have already been made using SR-FTIR. However, challenges remain, concerning the optical limitations not only of detectors but also the synchrotron infrared noises and instabilities; making the processing of spectrums more complicated and narrowing the application of this technology in practice, as large-scale scientific facilities with synchrotron radiation beamlines are frequently located away from hospitals, which makes it inconvenient for medical diagnosis.

With the development of infrared and microscope technology, nanospectroscopy is the emerging direction and future trend. By combining nano-infrared microscopy and spectroscopy with atomic force microscope imaging, the domain formation of these different polymer components can be identified and chemically detected [57]. And the infrared spectra of phospholipid monolayers with nanoscale spatial resolution can be obtained [58]. Now, the 2D information can easily be obtained in stem cells by SR-FTIR. But difficulties remain in 3D information acquirement. Thus, approaches to producing tissue and 3D cellular samples should be promoted [59]. With the development of fourth-generation storage ring facilities and greater stability coupled with superior spatial resolution, the high-energy photon source provides a brighter future for infrared spectroscopy. It is reasonable to believe that synchrotron infrared microspectroscopy deserves further investigation and development, especially in stem cells related studies.

## Figures and Tables

**Table 1:** Band assignment of major absorptions in SR-FTIR data of stem cells.

Number	Absorption Bands (cm^−1^)	Band Assignments	References
1	~1080	ν_s_(PO_2_^−^) of nucleic acids	[21,27,28,30,32,33]
2	1225–1238	ν_as_(PO_2_^−^) of nucleic acids	[25,27,28,30]
3	~1245	S-O stretching of proteoglycan	[14]
4	~1443	ν_as_(CH_3_) of protein	[30]
5	1550–1580	amide II	[21,30,32]
6	1630–1650	amide I	[21,30,32,36]
7	1714–1741	Ester, C=O stretching of nucleic acids and lipids	[17,29]
8	~2850	ν_s_(CH_2_)of lipids	[17,36]
9	~2923	ν_as_(CH_3_) of lipids	[17,36]

ν_s_: symmetric stretching vibration; ν_as_: asymmetric stretching vibration.

## Data Availability

Not applicable.
